# Paraoxonase 2 Protein Is Spatially Expressed in the Human Placenta and Selectively Reduced in Labour

**DOI:** 10.1371/journal.pone.0096754

**Published:** 2014-05-09

**Authors:** Samy Alwarfaly, Akrem Abdulsid, Kevin Hanretty, Fiona Lyall

**Affiliations:** 1 Institute of Medical Genetics, University of Glasgow School of Medicine, Yorkhill Hospital, Glasgow, United Kingdom; 2 Maternity Hospital, Southern General Hospital, Glasgow, United Kingdom; Medical Faculty, Otto-von-Guericke University Magdeburg, Medical Faculty, Germany

## Abstract

Humans parturition involves interaction of hormonal, neurological, mechanical stretch and inflammatory pathways and the placenta plays a crucial role. The paraoxonases (PONs 1–3) protect against oxidative damage and lipid peroxidation, modulation of endoplasmic reticulum stress and regulation of apoptosis. Nothing is known about the role of PON2 in the placenta and labour. Since PON2 plays a role in oxidative stress and inflammation, both features of labour, we hypothesised that placental PON2 expression would alter during labour. PON2 was examined in placentas obtained from women who delivered by cesarean section and were not in labour and compared to the equivalent zone of placentas obtained from women who delivered vaginally following an uncomplicated labour. Samples were obtained from 12 sites within each placenta: 4 equally spaced apart pieces were sampled from the inner, middle and outer placental regions. PON2 expression was investigated by Western blotting and real time PCR. Two PON2 forms, one at 62 kDa and one at 43 kDa were found in all samples. No difference in protein expression of either isoform was found between the three sites in either the labour or non-labour group. At the middle site there was a highly significant decrease in PON2 expression in the labour group when compared to the non-labour group for both the 62 kDa form (p = 0.02) and the 43 kDa form (p = 0.006). No spatial differences were found within placentas at the mRNA level in either labour or non-labour. There was, paradoxically, an increase in PON2 mRNA in the labour group at the middle site only. This is the first report to describe changes in PON2 in the placenta in labour. The physiological and pathological significance of these remains to be elucidated but since PON2 is anti-inflammatory further studies are warranted to understand its role.

## Introduction

Pregnancy is characterized by a complex interplay of inflammatory events regulated by both the innate and acquired immune systems. In humans parturition involves interaction of hormonal, neurological, mechanical stretch and inflammatory pathways and the placenta plays a crucial role [1]–[4]. The paraoxonases (PON) are multifaceted and pleiotropic enzymes encoded by three highly conserved genes (PON1, PON2, and PON3) located on chromo- some 7q21.3–22.1 [5]. They have multifunctional roles and are involved in various biochemical pathways. These include protection against oxidative damage and lipid peroxidation, modulation of endoplasmic reticulum stress, regulation of cell proliferation/apoptosis contribution to innate immunity and detoxification of reactive molecules and bioactivation of drugs [5]–[6]. Phylogenetic analysis has shown that PON1 and PON3 arose from gene duplication of the ancestral PON2 gene [5]–[6]. PON1 and PON3 are circulating proteins associated with high-density lipoproteins whereas PON2 is expressed in many tissues and is cell associated [7]. Research in the PON family has increased greatly in the last few years, particularly in the cardiovascular field [8].

Nothing is known about the role of PON2 in the placenta or whether it plays a role in labour. However since PON2 plays a role in oxidative stress and inflammation, both features of labour, we hypothesised that placental PON2 expression would alter during labour. Thus the aim of this study was to examine the spatial expression of PON2 in placentas obtained from women who delivered by cesarean section and were not in labour and to compare the expression of each zone with the equivalent zone of placentas obtained from women who delivered vaginally following an uncomplicated labour.

## Methods

### Ethics statement

Human term placentae were collected from pregnant women at the Southern General Hospital, Glasgow. All ethics protocols were followed as per Declaration of Helsinki. The study was approved by the West of Scotland research ethics service Signed patient consent was obtained prior to delivery. Patients were handed an information sheet telling them about the study before being handed the consent sheet. The information and consent sheets were also approved by the ethics committee. All signed consent sheets were stored incase of the need for audit.

### Patients studied

Placentae were collected from: (i) women who had uncomplicated pregnancies and delivered at term either vaginally (labour group, n = 6) or by caesarean section (non-labour group, n = 6). The labour group were all spontaneous labour and were a tight group (labour time minimum 3 hours maximum 8 hours). All placentas were free of infection, confirmed by the pathology report of every placenta. The non-labour group were all definitely without labour. All were planned Caesarean sections performed for obstetric reasons: breach presentation (2) previous caesarean section (2) or maternal request (2). The groups studied had no underlying maternal conditions such as hypertension, preeclampsia, diabetes or gestational diabetes or any other medical disorders. There was no fetal pathology such as fetal growth restriction. Patient demographics were compared with the student t-test. There was no significant difference in maternal age (non-labour 28.33±5.71 versus labour 26±2.28 years), placental weight (non-labour 594.7±110.5 versus labour 589.5±75 g), birth weight (non-labour 3443±537 versus labour 3719±347 g), number of primigravid (non-labour n = 2 versus labour n = 4) or number of smokers (n = 0 labour versus n = 2 non-labour).

### Placental sampling

For each patient (6 patients per group), placental samples (∼1 cm^3^) were obtained from three sites by taking measurements from the cord insertion point: inner third closest to cord insertion point (inner zone), middle of placenta (middle zone) and outer third of placenta (outer zone) of placenta. Within each zone four separate samples were obtained representing the four quadrants as previously described [9]–[11]. Placentas had a central cord insertion. Samples were rinsed and immediately flash frozen in liquid nitrogen. For this study we had performed a power analysis using G*Power 3.1 for Macintosh and based the numbers on previously published work with the same samples [9]–[11].

### Chemicals

All chemicals were purchased from Sigma-Aldrich (U.K.) unless stated otherwise.

### Tissue Homogenizing For Western Blot

Placental samples were recovered from storage at −70°C and ground to a fine powder in liquid nitrogen using a mortar and pestle. The frozen powder was then homogenised in the presence of protease inhibitors as previously described [9]–[11]. Placenta homogenates were spun at 5000 g for 10 min at 4°C to remove debris then supernatants were collected, divided into aliquots and stored at −70°C. Protein concentrations were determined by Bradford analysis using bovine serum albumin as a standard.

### Western Blotting

Western blotting was performed as described previously [9]–[11] with some modifications. A volume containing 50 µg of each sample was separated by SDS-PAGE electrophoresis on 10% sodium dodecyl sulfate-polyacrylamide resolving gels. Pre-stained low range molecular weight markers (BioRad, UK) were loaded onto each gel. Transfer of proteins to Hybond ECL nitrocellulose membranes (Amersham Pharmacia Biotech, UK) was performed at 22V and 200 mA for 30 min. Membranes were blocked in 5% goat serum (Serotec) in TBSTB buffer (20 mM TRIS pH 7.5, 0.5 M NaCl, 0.4% Tween and 0.25% bovine serum albumin) for 1 h at room temperature (RT). Primary antibodies were pre-absorbed in 5% human serum in TBSTB at RT during the blocking process. Membranes were incubated for 1 h at RT with primary antibody solution. The PON2 (mouse monoclonal antibody) was obtained from Santa Cruz Biotechonolgy, USA (catalogue number sc-373981) and used at concentration of 1∶200. Membranes were washed and then incubated for 1 h at RT with horseradish peroxidase conjugated goat anti-mouse secondary antibody (antibody (SantaCruz (sc-2005) diluted 1∶1000 in TBSTB. Membranes were rinsed with TBSTB (2×5 min) and once with distilled water. The same samples were exposed to a β-actin antibody (Sigma) to confirm even protein loading as shown previously [9]–[11]. Immunologically reactive proteins were visualised and quantified as described previously [9]–[11]. A standard curve was performed for different blot exposures and densitometry was performed when bands were on the linear part of the loading graph as described previously [9]–[11]. For each group of experiments the same loading control placenta sample was added to every gel and the densitometry units were normalized to that. We previously confirmed that this method of analysis gives similar findings to other quantitative methods of densitometry [9]–[11].

### Quantitative Real Time-Polymerase Chain Reaction (RT-PCR)

Total RNA was isolated using the RNeasy Midi Kit (Qiagen, 75142). RNA (100 ng) was reverse transcribed into cDNA. Buffers and primers were obtained from the QuantiTect Kit (Qiagen, 205310) and GoScript reverse transcriptase from Promega (A501C). PON2 expression was analyzed by RT-PCR using validated TaqMan Gene Expression assays with StepOnePlus (Applied Biosystems). β-actin was used as an endogenous control. A positive control human placenta cDNA (Primer design) was used. The relative target gene levels were calculated by comparative C_T_ (ΔΔC_T_).

### Statistical analysis

Patients details were compared using the student t-test. Statistical analysis was performed using Graphpad prism 5 on a PC. For Western blot analysis and RT-PCR analysis statistical analysis was performed using the Friedman test for non-parametric data.

## Results

### Experiment 1

**Figure 1:**
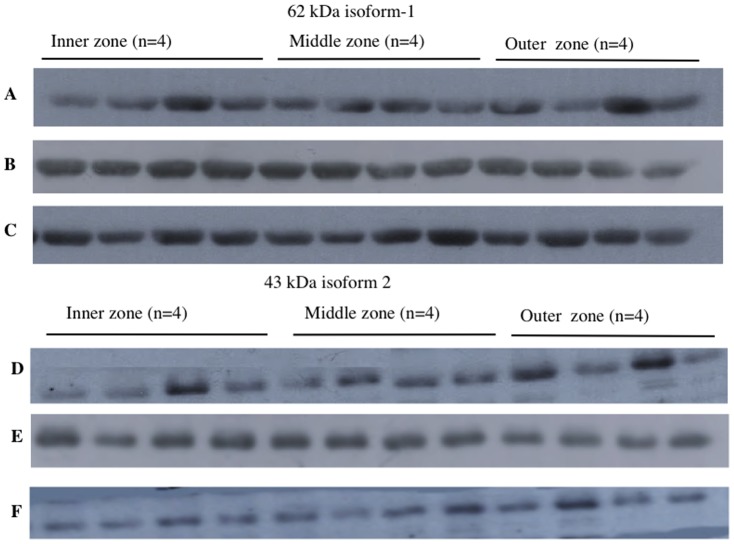
Western blots showing PON2 expression in inner, middle and outer zones of three individual placentas (non-labour group). Four quadrants were sampled in each zone. A, B and C show the 62Figure 1. D, E and F show the 43 kDa isoform 2 for the 3 placentas also shown in Figure 1.

**Figure 2:**
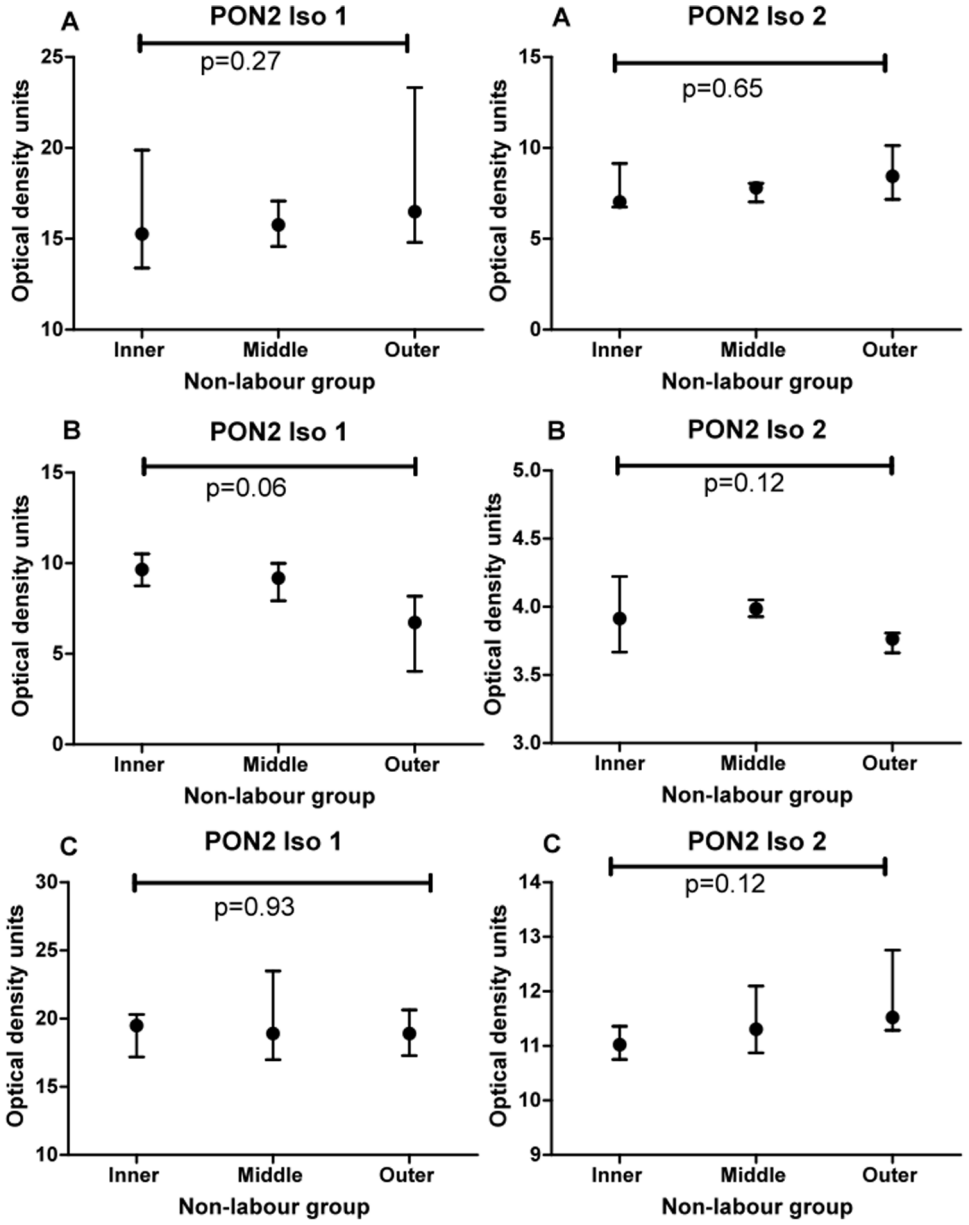
Graphs show median and interquartile range for PON2 isoforms 1 and 2 for the combined 4 quadrants sampled in the inner, middle and outer zones of three different placentas (A, B, C non-labour group) shown in Figure 1. Comparison between zones was performed using Friedman analysis.

This experiment was designed to test if there was a spatial difference in expression of PON2 within individual placentas obtained from women who were not in labour. Two main bands were detected in all placenta samples, one at 62 kDa and one at 43 kDa. These are named as isoform 1 62 kDa and isoform 43 kDa throughout. Examples of Western blots showing PON2 (isoform 1, 62 kDa) expression for 3 different placentas (all non-labour) are shown in Figure 1 (blots A-C). Examples of Western blots showing PON2 (isoform 2, 43 kDa) expression for the same three placentas are also shown in Figure 1 (blots D–F). Friedman test analysis showed there was no difference in expression of either isoform between the three sites (inner, middle, outer) within individual placentas. The graphs for blots A–C (isoform 1, 62 kDa) are shown in Figure 2 (A–C) and the graphs for blots D–F (isoform 2, 43 kDa) are shown in Figure 2 (D–F).

### Experiment 2

**Figure 3:**
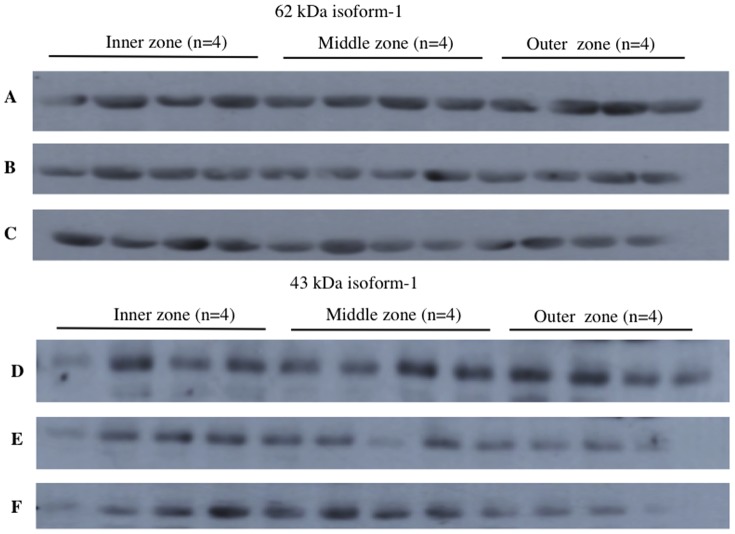
Western blots showing PON2 expression in inner, middle and outer zones of three individual placentas (labour group). Four quadrants were sampled in each zone. A, B and C show the 62 kDa isoform 1. D, E and F show the 43 kDa isoform 2.

**Figure 4:**
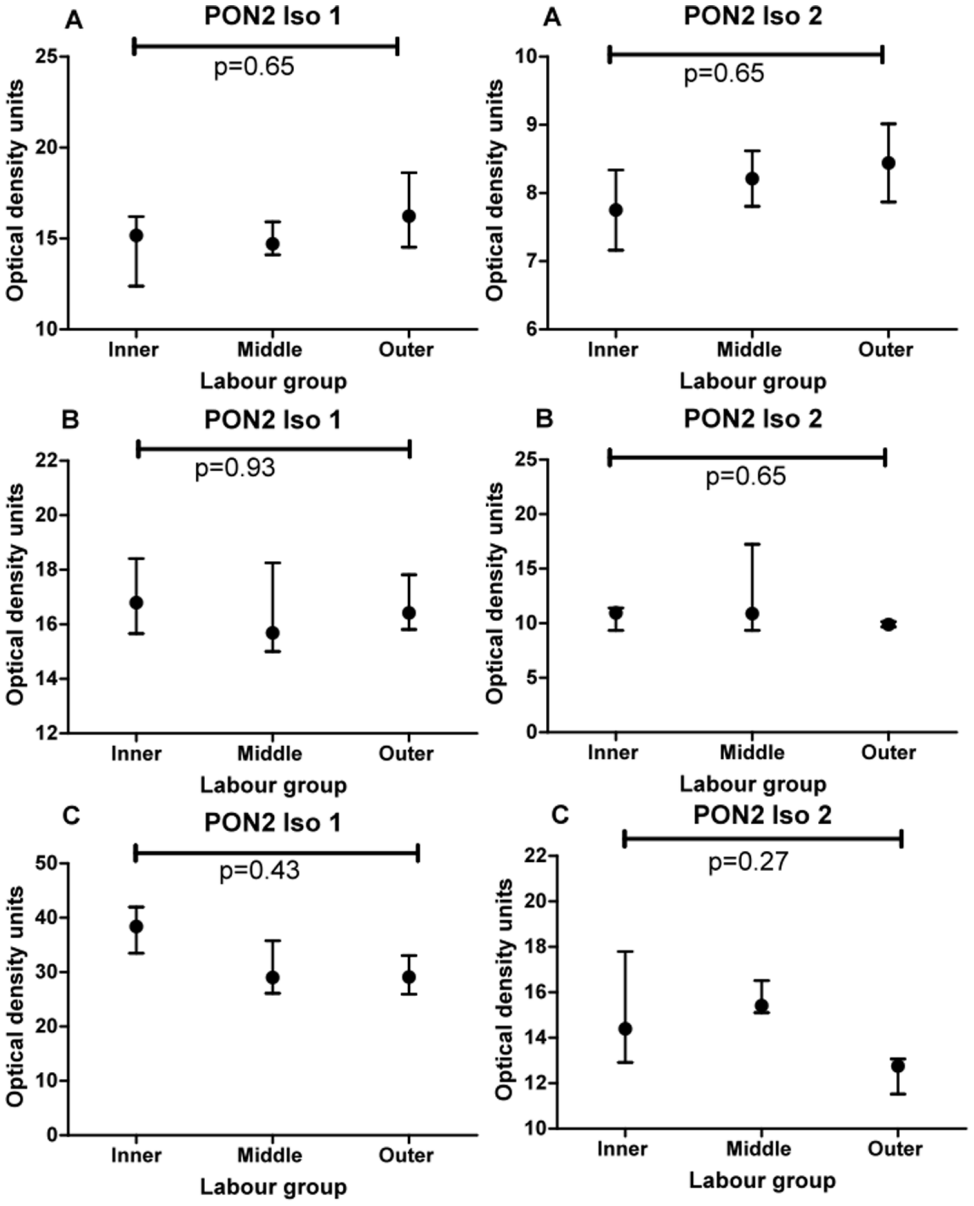
Graphs show median and interquartile range for the 4 quadrants sampled in the inner, middle and outer zones of each of the three placentas (labour group) shown in Figure 3. Comparison between zones was performed using Friedman analysis.

This experiment was designed to test if there was a spatial difference in expression of PON2 within individual placentas obtained from women who were in labour. Two isoforms (62 kDa and 43 kDa) were also identified in all placenta samples. Examples of Western blots showing PON2 (isoform 1, 62 kDa) expression for 3 different placentas (labour) are shown in Figure 3 (blots A–C). Examples of Western blots showing PON2 (isoform 2, 43 kDa) expression for the same three placentas are also shown in Figure 3 (blots D–F). Friedman test analysis showed there was also no difference in expression of either isoform between the three sites (inner, middle, outer). The graphs for blots A–C (isoform 1, 62 kDa) are shown in Figure 4 (A–C) and the graphs for blots (isoform 2, 43 kDa) are shown in Figure 4 (D–F). In summary for experiment 1 and 2, there was no spatial difference in expression of either PON2 isoform within individual placentas both in non-labour and in labour groups.

### Experiment 3

**Figure 5:**
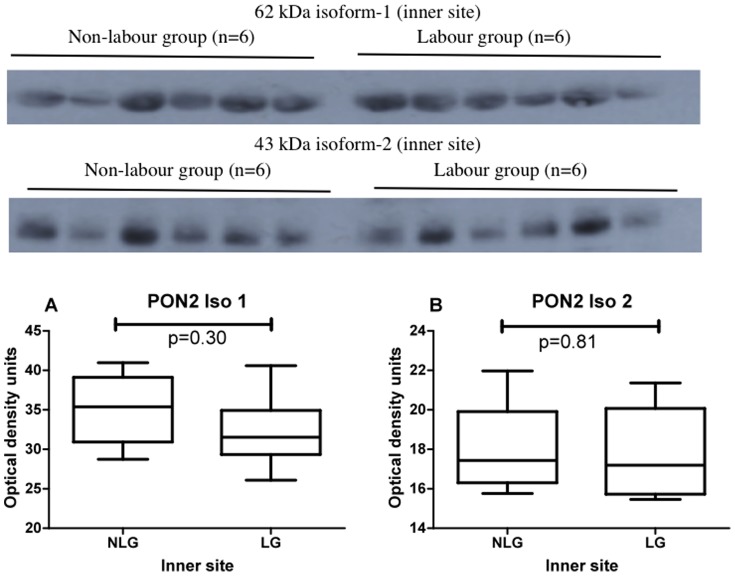
Western blots showing PON2 62(upper panel) and PON2 43 kDa isoform 2 (lower panel) expression in the inner placental site in non-labour and labour (n = 6 in each group). The graphs show the box and whiskers analysis of the blots. Comparison between zones was performed using Friedman analysis. Graph A, 62; Graph B, 43 kDa isoform 2. NLG non-labour group, LG labour group.

**Figure 6:**
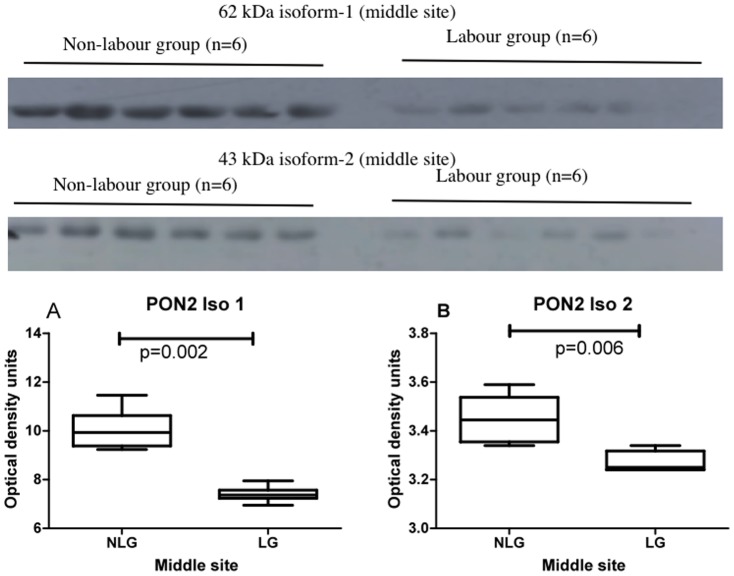
Western blots showing PON2 62(upper panel) and PON2 43 kDa isoform 2 (lower panel) expression in the middle placental site in non-labour and labour (n = 6 in each group). The graphs show the box and whiskers analysis of the blots. Comparison between zones was performed using Friedman analysis. Graph A, 62; Graph B, 43 kDa isoform 2. NLG non-labour group, LG labour group.

**Figure 7:**
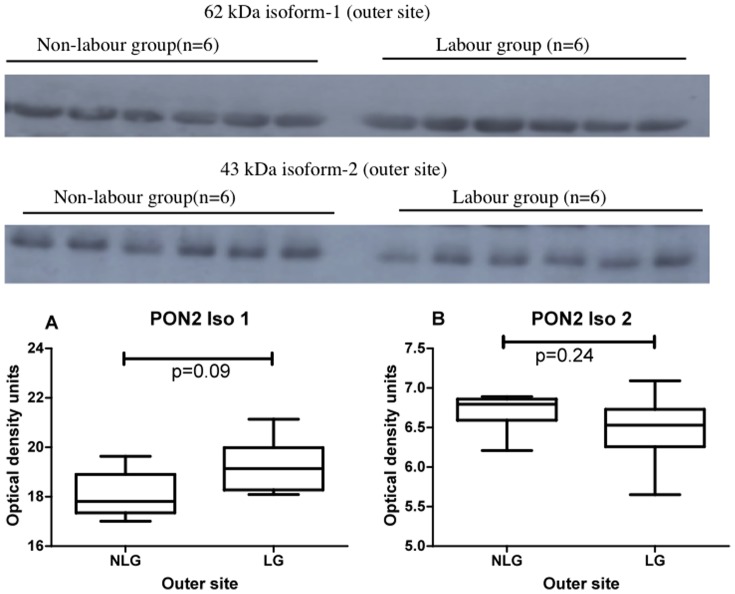
Western blots showing PON2 62(upper panel) and PON2 43 kDa isoform 2 (lower panel) expression in the outer site in non-labour and labour (n = 6 in each group). The graphs show the box and whiskers analysis of the blots. Comparison between zones was performed using Friedman analysis. Graph A, 62; Graph B, 43 kDa isoform 2. NLG non-labour group, LG labour group.

This experiment was designed to test if there was any difference in PON2 expression between labour and non-labour groups at the inner, middle or outer areas of placentas. Western blots showing placental PON2 expression in non-labour and labour at the inner site are shown in Figure 5 (upper panel 62 kDa isoform 1; lower panel 43 kDa isoform 2). The graphs and statistical analysis are shown below the blots. No differences were found. Western blots showing placental PON2 expression in non-labour and labour at the middle site are shown in Figure 6 (upper panel 62 kDa isoform 1; lower panel 43 kDa isoform 2). The graphs and statistical analysis are shown below the blots. There was a highly significant decrease in PON2 expression in the labour group when compared to the non-labour group for both the 62 kDa isoform 1, (p = 0.02) and the 43 kDa isoform 2, (p = 0.006). Western blots showing placental PON2 expression in non-labour and labour at the outer placental site are shown in Figure 7 (upper panel 62 kDa isoform 1; lower panel 43 kDa isoform 2). The graphs and statistical analysis are shown below the blots. No differences were found.

**Figure 8:**
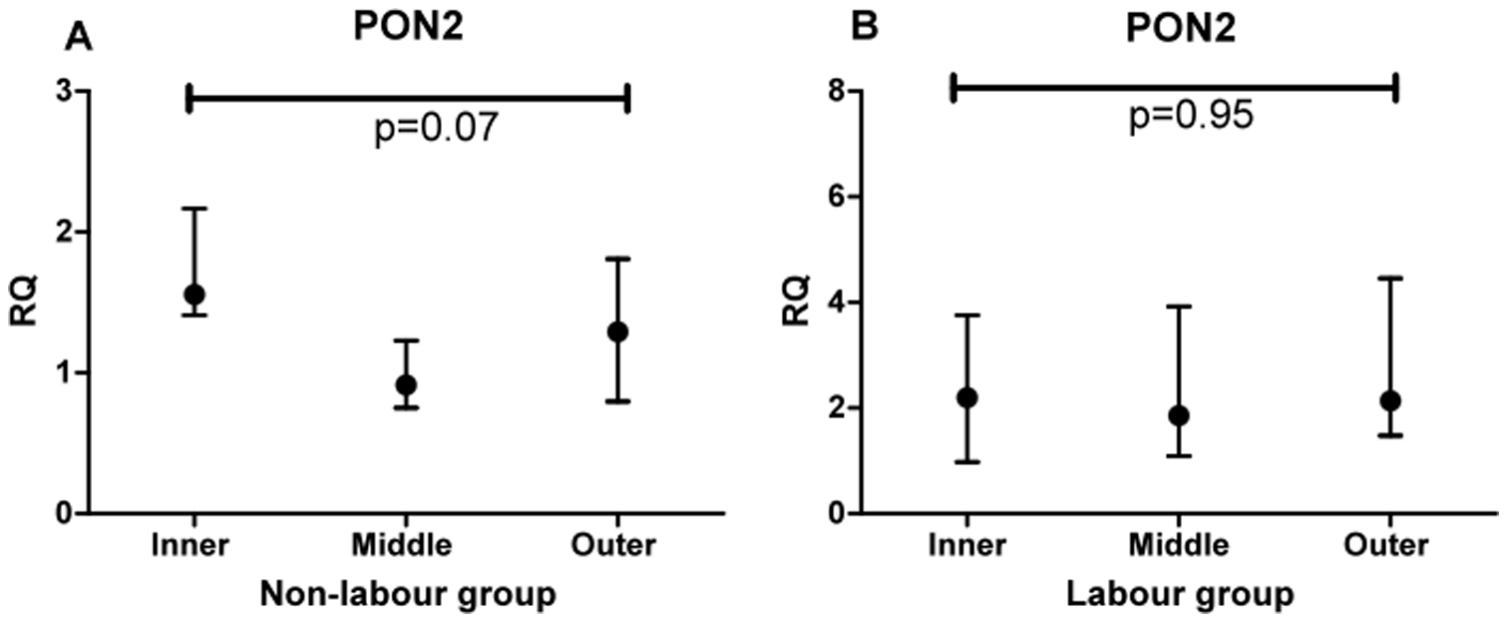
RQ values for mRNA measurements in inner, middle and outer placental sites within individual placentas. A non-labour and B labour. Comparison between zones was performed using Friedman analysis.

Experiment 4 was designed to test if there were any differences in PON2 mRNA expression within individual placentas at different zones in either labour or non-labour. Figure 8 shows the PON2 RQ values in the inner, middle and outer zones for non labour (A) and labour (B). Just as for both PON2 protein isoforms, no spatial differences were found at the mRNA level in either labour or non-labour.

**Figure 9:**
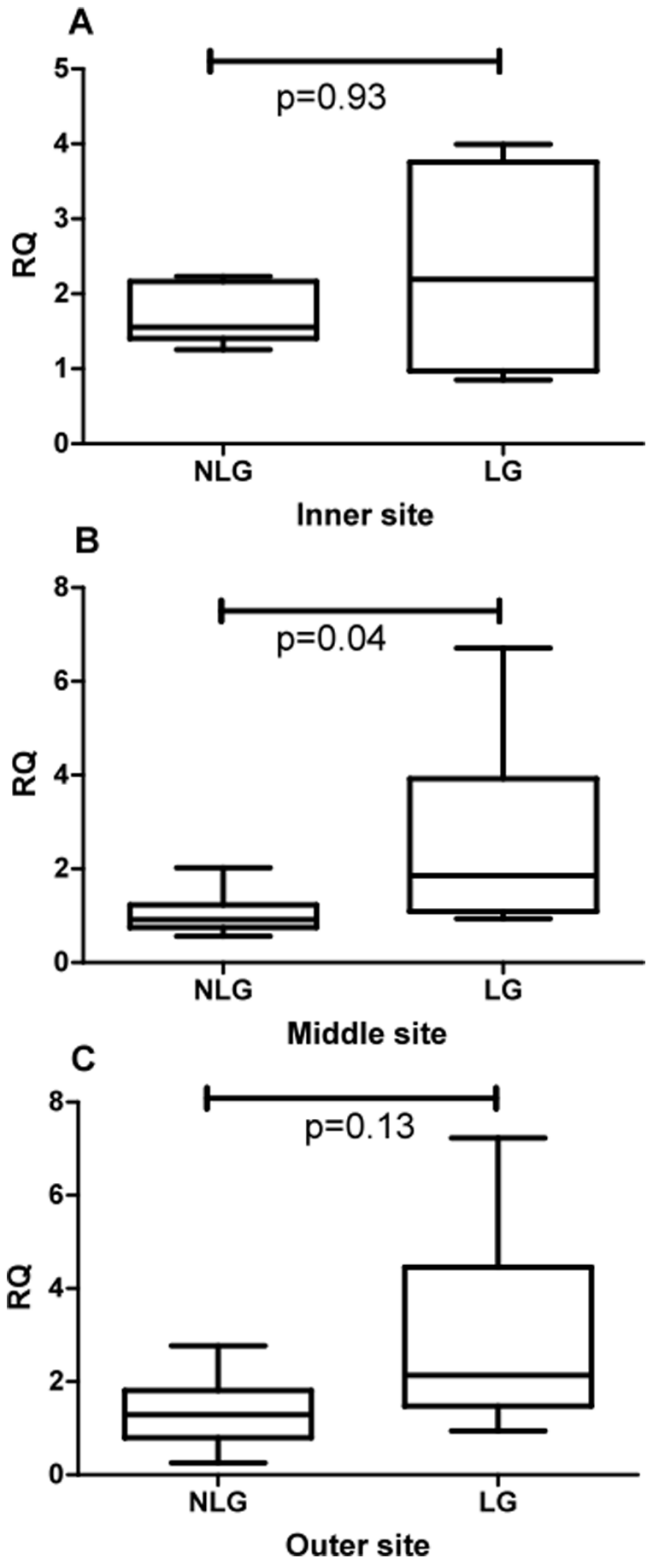
RQ values for mRNA measurements in inner, middle and outer placental sites for labour compared with non-labour. Comparison between zones was performed using Friedman analysis.

The final experiment 5 tested whether there was any difference between labour and non-labour at the inner, middle our outer sites at the mRNA level. The results are shown in Figure 9. As for PON2 protein no differences were found between labour and non-labour at the inner or outer placental sites. There was, paradoxically, an increase in PON2 mRNA in the labour group at the middle site.

## Discussion

### PON2 and present findings

The PON family form dimers and trimers and can be glycosylated; this may, in part, explain the range of different molecular weights reported to date [12]. In the present study two main bands were detected with the PON2 antibody. The smaller band of around 43 kDa is the range reported for PON2. The larger band around 62 kDa has been described in liver lysates; reported in the PON2 antibody datasheet (http://www.novusbio.com/PON2-Antibody_NBP1-52005.html). This larger band was successfully blocked by incubation with the immunizing peptide showing it is a form of PON2. The reduced placental expression of PON2 protein expression found in labour in the middle site would be likely to promote inflammatory processes. Thus it is possible that a reduction in PON2 protein may help to initiate or promote labour but this is speculative. Why PON2 mRNA was found to be increased may seem paradoxical but it may be that the increased mRNA is a separate response to oxidative stress as a result of myometrial contractions. The reduction in PON2 protein could also be due to processes other than reducing mRNA synthesis, such as increased protein degradation. PONs are glycosylated with high-mannose-type sugars, which are important for protein stability but are not essential for their enzymatic activities [12]. Whether alterations in glycosylation of PON2 could affect PON2 expression in the placenta would require future research.

### Link to oxidative stress and ischemia-reperfusion injury

Oxidative stress occurs when the production of reactive oxygen species overwhelms the intrinsic anti-oxidant defenses. At the moment it is not clear whether the observed reduction in PNO2 protein expression in the placenta in the labour group is a consequence of labour itself or, alternatively, contributed to labour via endocrine, hemodynamic or other processes. Since human patients have been used it is difficult to separate these effects. It is known however that contraction of the uterus leads to ischemic-reperfusion injury that can alter placental protein expression [13]. Furthermore Doppler ultrasound studies have demonstrated an inverse relationship between uterine artery resistance and the intensity of uterine contractions during labour [14]. It has been shown in chronically instrumented pregnant rhesus monkeys that placental blood flow is almost completely stopped during sustained myometrial contractions and that this occurs as a result of compression of the arcuate and spiral arteries [15], [16]. The closest human model to this was performed on patients prior to termination of pregnancy at 17–20 weeks of gestation [17]. During oxytocin-induced contractions, a 50% reduction in flow into the intervillous space, as well as a fall in entry sites and volume, was found compared to when no contractions occurred. This suggests that intermittent perfusion of the intervillous space would lead to an ischemic-reperfusion injury of the placenta. Reactive oxygen species and the oxidant/antioxidant balance can be affected as a result [17]. In keeping with this labour has been reported to be associated with placental alterations in several pathways linked to oxidative stress [18]–[24] Other studies looking at heat shock proteins, Mn-SOD, Cu/Zn-SOD and peroxidation of lipids also show an association between labour and placental oxidative stress [9]–[10], [13].

The biochemical events associated with labour involve increased interleukin-1β and prostaglandin synthesis [24]. The later stages of gestation are likely to be associated with more fluctuations in blood flow as demand by the placenta and fetus is maximal. COX-2 increases in mouse and rat placental trophoblast with gestation [25]–[26]. In vitro studies also suggest that hypoxia-reoxygenation increases COX-2 which may in turn play a role in augmentation or even initiation of labour [26]. Furthermore human placental trophoblast show activation of the NF-κB and COX-2 during labour. Increase in levels of cleaved caspase-3 and cleaved caspase-9 confirm evidence of placental apoptosis during labour [13]. As will be discussed below PON2 expression and activity can affect these biochemical events.

### PONS

The paroxonases were named so because the substrate for PON1 is paroxon which is the active metabolite of the organophosphorus insecticide parathion. PON2 and 3 lack this esterase activity despite the similar nomenclature. PON1, 2 and 3 are lactonases and PON2 has the highest activity of the three PONs [5], [12], [27]–[29]. PON1 is mainly synthesized by the liver. It associates with high-density lipoprotein (HDL) in the circulation [30]. PON1 hydrolyses several substrates; these include organophosphate insecticides and nerve gases, lipid hydroperoxides, lactones and thiolactones [12], [31]–[33]. PON1 is a potent anti-atherosclerotic enzyme [34]–[38]. PON1 and PON3 proteins are present in plasma and reside in the high-density lipoprotein fraction and protect against oxidative stress by hydrolyzing certain oxidized lipids in lipoproteins, macrophages, and atherosclerotic lesions [37]–[40]. Paraoxonases are important detoxifying and anti-oxidative enzymes with roles being described in organophosphate poisoning, diabetes, obesity, cardiovascular diseases, innate immunity and with atherosclerosis [41]–[45]

### PON2 and cell death

Endoplasmic reticulum (ER) stress activates the unfolded protein response (UPR) pathway and pro-apoptotic CHOP protein in the presence of overwhelming ER stress, [46]–[48]. Mitochondria also play a key role in cell death via production of excess reactive oxygen species (ROS) [49]. It has been shown that human PON2 diminished not only ROS but also ER stress-induced apoptosis in vascular cells [50]. PON2 is expressed in several tissues with antioxidant properties. It is capable of preventing cell-mediated oxidative modification of low density lipoprotein and ER stress-induced apoptosis. [7], [9], [51]. PON2 is not present in serum lipoprotein fractions but exists as an intracellular protein found in almost every tissue, particularly at the perinuclear region, ER and mitochondria. [7], [50], [52]. Natural substrates remain unknown albeit PON2, as part of the innate immune system, appears involved in the defense against infections by the human pathogen *Pseudomonas aeruginosa* [52]. Several studies demonstrated that PON2 protected macrophages, vascular and other cells against oxidative stress, whereas its downregulation reversed this effect [7], [50], [53].

PON2 has been shown to be overexpressed in several cancers and it has been suggested that this may be due to the fact that PON2 confers resistance to apoptosis as well as oxidative stress [54]. It has been shown that during ER stress, high levels of PON2 lowered redox-triggered induction of pro-apoptotic CHOP particularly via the JNK pathway, which prevented mitochondrial cell death signaling [54]. Apart from CHOP, PON2 also diminished intrinsic apoptosis as it prevented mitochondrial superoxide formation, cardiolipin peroxidation, cytochrome *c* release, and caspase activation. Oxidized lipids can also induce proinflammatory genes, such as TNF-α and MCP-1, via NF-κB activation [55]. Therefore one possibility is that less placental PON2 would result in more oxidized lipids and more NF-κB activation which would promote labour. Macrophages harvested from PON2-/- mice are more susceptible to cellular oxidative stress than wild-type macrophages [56]. PON2 also inhibits the development of atherosclerosis in mice, via mechanisms involving the reduction of oxidative stress [30], [45], [57]. One study investigated the distribution of PON1 and 2 mRNA in 24 human tissues, using gene expression panels. PON2 but not PON1 was identified in placenta [58].

The mechanism by which PON2 modulates ROS production is still unclear [59]–[62]. Lactones have been suggested to be the natural substrates of PON2 and PON2 lactonase activity has been shown to correlate with this enzyme's biological antioxidant properties.

### Genetics studies

Studies have shown increased risk of coronary artery disease, carotid atherosclerosis and stroke in patients with low paraoxonase activity PON1 and 2 alleles [63]. Specific gene polymorphisms for PON1 and PON2 have been reported in children born pre-term. [63]. There is a lack of data in this new field. However term labour results from a physiological reduction in the influence of endocrine signals and other factors that act to inhibit myometrial contractility, in conjunction with the activation of pro-inflammatory biochemical pathways that precede myometrial activation and contraction. Premature activation of inflammatory mediators is a major feature of the pathophysiology of preterm labour and in particularly very preterm labour where it is often induced by infection [3]. Whether PON2 may be involved in pre-term labour is worthy of investigation.
